# Biomarkers of Immersion in Virtual Reality Based on Features Extracted from the EEG Signals: A Machine Learning Approach

**DOI:** 10.3390/brainsci14050470

**Published:** 2024-05-07

**Authors:** Hamed Tadayyoni, Michael S. Ramirez Campos, Alvaro Joffre Uribe Quevedo, Bernadette A. Murphy

**Affiliations:** 1 Faculty of Health Sciences, Ontario Tech University, Oshawa, ON L1G 0C5, Canada; hamed.tadayyoniahrab@ontariotechu.net; 2 Faculty of Biomedical Engineering, Universidad Escuela Colombiana de Ingeniería Julio Garavito, AK 45 #205-59, Bogotá 111166, Colombia; michael.ramirez@mail.escuelaing.edu.co; 3 Faculty of Business and Information Technology, Ontario Tech University, Oshawa, ON L1G 0C5, Canada; alvaro.quevedo@ontariotechu.ca

**Keywords:** virtual reality, immersion, task difficulty, electroencephalography (EEG), biomarkers, machine learning

## Abstract

Virtual reality (VR) enables the development of virtual training frameworks suitable for various domains, especially when real-world conditions may be hazardous or impossible to replicate because of unique additional resources (e.g., equipment, infrastructure, people, locations). Although VR technology has significantly advanced in recent years, methods for evaluating immersion (i.e., the extent to which the user is engaged with the sensory information from the virtual environment or is invested in the intended task) continue to rely on self-reported questionnaires, which are often administered after using the virtual scenario. Having an objective method to measure immersion is particularly important when using VR for training, education, and applications that promote the development, fine-tuning, or maintenance of skills. The level of immersion may impact performance and the translation of knowledge and skills to the real-world. This is particularly important in tasks where motor skills are combined with complex decision making, such as surgical procedures. Efforts to better measure immersion have included the use of physiological measurements including heart rate and skin response, but so far they do not offer robust metrics that provide the sensitivity to discriminate different states (idle, easy, and hard), which is critical when using VR for training to determine how successful the training is in engaging the user’s senses and challenging their cognitive capabilities. In this study, electroencephalography (EEG) data were collected from 14 participants who completed VR jigsaw puzzles with two different levels of task difficulty. Machine learning was able to accurately classify the EEG data collected during three different states, obtaining accuracy rates of 86% and 97% for differentiating easy versus hard difficulty states and baseline vs. VR states. Building on these results may enable the identification of robust biomarkers of immersion in VR, enabling real-time recognition of the level of immersion that can be used to design more effective and translative VR-based training. This method has the potential to adjust aspects of VR related to task difficulty to ensure that participants are immersed in VR.

## 1. Introduction

Virtual reality (VR) allows the delivery of novel solutions in various domains such as entertainment [1], simulations [2], tele-rehabilitation [3,4], and training [5]. In particular, VR training applications not only provide the opportunity to experience scenarios that impose high physical or hygienic risks [6], but also allow trainees to practice the module as many times as necessary without being limited by fear of wasting real resources [7]. Despite its potential, VR’s limitations include physical drawbacks such as VR-induced motion sickness [5] and the weight of the head-mounted device (HMD) [8]. Furthermore, VR-based training may not accurately simulate the level of tactile, haptic, or proprioceptive feedback with which users need to be trained to develop the required kinaesthetic skills [9]. Additionally, virtual environments may fail to accurately represent the real-world scenario in terms of visual and auditory cues and fidelity [10]. These restrictions may decrease the level of effectiveness of VR-based training and must be studied and addressed to optimize VR for training in certain applications [5].

As a result of these limitations, the success of VR training can depend on how successful it is in engaging the user’s senses and cognitive capabilities to the same level as its real-world counterpart. In the literature, engagement is defined in terms of different quantities such as presence, flow, fidelity, and immersion [11]. Flow is defined as the process of optimal experience [12], presence refers to the psychological sense of being in the virtual environment [13], and immersion is defined as the degree to which the user feels engaged and absorbed in the environment and attends to the planned task [14]. Immersion encompasses different aspects of the sense of ‘being there’ [15], including being caught up in the sensory input of the virtual environment, as well as being mentally and cognitively invested in the intended task. Immersion that refers to the sensory information received by the user from the virtual environment is called sensory immersion [16], while cognitive immersion is defined by the degree of engagement of the user caused by the task’s demands [1]. Although the former is mostly constrained by technology-related aspects of the virtual environment and how well the software and hardware provide the required levels of different real-world sensory information [4], the latter is dependent on how much the designed task engages the user [17]. Immersion provides a better quantification of engagement in the evaluation of a virtual training designed to replicate the real-world experience, as its definition encompasses both sensory and cognitive components of VR training [11].

Research on immersion has been crucial to determine the impact and success of VR experiences in the translation of cognitive and motor learning [18]. There are different subjective and objective methods proposed in the literature to study immersion. Subjective methods strongly rely on participants’ opinions and self-reported data [13,19] while considering the sense of immersion tied to the phenomenological experience of the user [1]. These measures rely on the understanding that the user has of the concept of immersion [19] and are impacted by the inherent subjectivity of the measured quantity. Additionally, asking about immersion while the user is inside the virtual environment breaks the immersion, as it distracts the user from their subjective experience [20], and asking about it afterwards makes the results highly dependent on the recollection of the user’s experience [21]. Therefore, quantifying immersion in a consistent and objective manner that enables researchers to compare their findings and investigate the difference between immersion levels resulting from different tasks, environments, levels of difficulty, circumstances, etc., is necessary. Researchers have investigated various objective methods of measuring immersion that do not require conscious deliberation from the participants [11,22], using performance-based and physiological-based points of view. Physiological measures have included eye tracking [11], galvanic skin response [23], electrocardiogram [24], and electroencephalography (EEG) [2,25], among others.

In the literature, to our knowledge, the use of EEG for studying immersion has been limited to measuring the amplitude of event-related potentials (ERPs), evoked in response to a stimulus that is not related to the task in which the immersion of the participant is studied. This is followed by a statistical analysis of ERP amplitudes to study the differences between different levels of immersion and/or presence [1,2,23,25,26]. Although this method is more promising than other physiological measures in terms of accuracy and resistance to confounding variables (including being influenced by how virtual environments represent information, boredom, and exhaustion), it still lags in offering a robust marker for identifying immersion that is not influenced by potential confounding variables, and it has resulted in heterogeneous, and in some cases contradictory findings [1,25]. It also suffers from an inability to identify and differentiate low and high levels of immersion in real time. Machine learning (ML) methods for classifying EEG signals can offer the ability to differentiate between different levels of immersion in real time.

In the literature, EEG-based machine learning and other classification approaches have been used in various paradigms to extract insightful meaning from different mathematical features of the signals. Kamińska et al. [27] and Aliyari et al. [28] were able to classify different levels of stress imposed on the users in the virtual environment. Deep learning has been used to extract information from EEG for stroke patients performing a real-time rehabilitation experiment [29]. Moncada et al. proposed a method for a VR-based protocol to classify important characteristics related to epilepsy [30], while Yildrim has reviewed ML-based methods used to classify EEG characteristics attributed to cybersickness [31]. Hekmatmanesh et al. investigated the use of different methods based on EEG (based on a common spatial pattern algorithm) to improve the detection of motor imagery patterns in EEG signals in brain–computer interface applications by evaluating the efficiency of various types of classifiers [32]. Other work has investigated the possibility of using brain–computer interfaces to control movements in VR based on ML-based movement prediction [33], and other work has investigated the applications of machine learning approaches for EEG-based emotion recognition [34].

These studies show that the potential for extracting relevant features for classification of EEG recordings is promising, with the potential to identify biomarkers of sensory processing in EEG recordings of a VR-based task. These methods introduce more robust biomarkers for their corresponding applications, where more accurate and homogeneous results are obtained, but also offer the potential for automatic recognition and classification of EEG data in real time. If they can progress to real-time measurement, machine learning approaches have the potential to address the limitations of VR-based training on the performance and transfer of skills to the real world and contribute to improving the design of VR-based training. Additionally, ML approaches might enable real-time customization of various features of training according to the individual characteristics of a user.

In this study, immersion was attributed to the level of difficulty of the task, based on the past literature [35,36]. Therefore, different levels of task difficulty were used, which included sitting idle and solving a jigsaw puzzle in easy and hard conditions in VR, where the number of pieces determined the difficulty of the task. Machine learning algorithms (stochastic gradient descent (SGD), support vector classifier (SVC), decision tree (DT), Gaussian naive Bayes (GNB), k-nearest neighbors (KNN), random forest (RF), and a multilayer perceptron (MLP)) were used to classify the EEG signals recorded during these states. Various temporal, frequency-domain, and non-linear features were used for analysing the EEG signals and in total two sets of features were tested (10 features for three or nine central channels and four frequency bands). The combination of a novel design protocol (which has shown its robustness in a recent study [25]) and machine learning approaches was used in the current study. The study aimed to determine whether machine learning approaches could accurately classify the three states based on the features extracted from EEG data, in addition to determining which features best represent different states of immersion.

## 2. Materials and Methods

### 2.1. Overall Experimental Procedure

A total of 14 right-handed individuals (7 male, 6 female, 1 preferred not to say) between the ages of 18 and 35 participated in this study. The dominance of the right hand was determined by a score of above 40 in the Edinburgh handedness inventory [37]. The study exclusion criteria required all participants not to have any neurological conditions (such as epilepsy, multiple sclerosis, skull fracture or serious head injury, attention deficit hyperactivity disorder, etc.), and not to have recurrent or chronic neck pain, and not to take any tricyclic antidepressants, neuroleptic or antipsychotic medications, or recreational drugs, as they can alter EEG suitability. Furthermore, to avoid hearing and severe visual conditions as well as motion sickness, which could compromise the results, the participants were asked if they had hearing problems, stereo blindness, or had reported previous VR-induced motion sickness; participants reporting any of these were excluded from the study. This study was approved by the research ethics board of the University of Ontario Institute of Technology (Ontario Tech University) (REB #17351).

Prior to the main study, we conducted various preliminary studies [1,2,3,23,26] and developed a protocol [25] to investigate the feasibility of the chosen task for discriminating between low and high levels of immersion. A VR jigsaw puzzle was selected for the study because it enables potential confounding variables, not related to immersion, to be minimized. This is described in greater detail below (Section 2.2).

**Figure 1:**
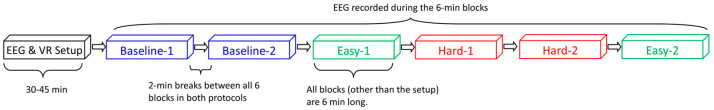
Overall experimental protocol.

The main study started with a calibration stage in which participants sat on a chair and wore both the EEG cap and the Meta Quest Pro VR headset. The calibration focused on collecting a ‘baseline’ data set with the participants watching a 360° pre-recorded video of the real study room while remaining idle for two 6 min blocks. After completing the baseline collection, the participants played through the jigsaw puzzles for four 6 min blocks of easy, hard, hard, and easy levels. The overall experimental protocol is depicted in Figure 1. The participants were instructed to use controllers to select, pick, reorient, and place pieces. The participants were allowed to interact with the game through a familiarization block with the objective of reducing the cognitive load that would be required when familiarizing with the controllers while solving the puzzle at the same time. A short 2 min break was anticipated in which the headset (and not the EEG cap) was removed, enforced to avoid exhaustion from wearing the headset, which weighs 722 g.

**Figure 2:**
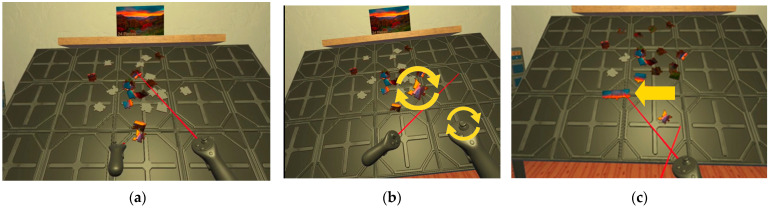
‘Jigsaw Puzzle VR’ game interactions: (**a**) Picking up puzzle pieces by pointing and selecting them using the trigger button; (**b**) rotating the puzzle piece with the thumb sticks; (**c**) the pieces are joined together when matched.

The ‘Jigsaw Puzzle VR’ (available through https://www.meta.com/experiences/5080756015327836/?utm_source=altlabvr.com (accessed on 9 July 2023)) game was chosen because it provided the closest experience to solving a puzzle in real life. This game allows users to use the controllers to move and put together the pieces (Figure 2). In this case, difficulty refers to how complex it is to complete the puzzle according to the number of pieces and the time required to complete the puzzle [25]. Two levels of difficulty were chosen: one with 24 pieces, set as easy difficulty; and a 60-piece puzzle selected for the hard difficulty. Each component of this procedure is defined in detail in the following subsections.

### 2.2. Choice of the Experimental Task

**Figure 3:**
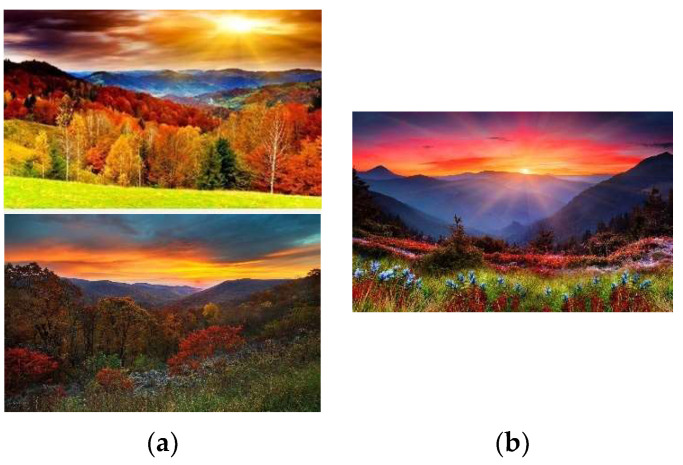
Photos of similar landscapes used for 2 difficulty levels of the jigsaw puzzle game: (**a**) used for the easy level and (**b**) used for the hard level. To have control over the difficulty level of the puzzles, the photos were chosen to resemble the same color distribution and scenery, so that the only difference between the levels was the number of the pieces chosen for each level of difficulty. (photo sources: ((**a**)—top) image from wallpapers.com, “Beautiful Scenery Trees Wallpaper”, accessed on 13 October 2023, © 2023 wallpapers.com; ((**a**)—bottom) Peakpx, “Shenandoah National Park”, accessed on 13 October 2023, © 2023 peakpx.com; (**b**) Peakpx, “view nature, bonito, flowers”, accessed on 13 October 2023, © 2023 peakpx.com).

Our proposed protocol employing a jigsaw puzzle provides a suitable testbed with the following highlights:

- The similarity between the easy and hard levels in terms of interactions highlights that the main difference between the difficulty levels is only related to the cognitive demand. The scenes for the easy and hard puzzles were chosen from very similar natural and ‘unfamous’ landscapes, similar in color and pattern, so that the participants were not stimulated by possible memories, emotions, and thoughts induced by other types of pictures. The images used for different blocks of playing the jigsaw puzzle are presented in Figure 3.
- The number of pieces for the puzzles was adjusted in our pilot studies to ensure that the easy and hard puzzles could be completed within the allocated study time. Furthermore, ensuring that the puzzle can be completed minimizes the risk of participants feeling demotivated, according to the motivational intensity model (MIM) [38]. Therefore, during the pilot phase of the study, several permutations of duration and number of pieces were tested to find the optimum combination [25]. We came up with the final number of pieces for easy and hard levels through multiple rounds of piloting in which different skilled and unskilled participants played the game with different number of pieces, puzzle scenes, and lengths. We tested durations as short as 3 min and as long as 12 min, together with the number of pieces as low as 20 pieces and as high as 96 pieces. Most participants could complete two easy puzzles (each with 24 pieces) or one hard puzzle (with 60 pieces) in the two 6 min blocks allocated to each condition.

### 2.3. Choice of Rest State (Baseline Collection)

During baseline data collection, the participant wears the VR HMD on top of the EEG cap. Additionally, the headset is powered during the baseline collection to have all possible confounding parameters caused by wearing the HMD exactly consistent between the easy and hard difficulties. Acknowledging that visual cues can influence cognitive load, we explored using a 180° version of the fixation cross (e.g., reticle) [39] in VR, and playing a 360° video of the same environment where the visual stimuli matched the same environment in which the participant was currently in. The 360° video was chosen over the fixation cross, since participants found that the latter was boring and monotonous, creating mental distractions that could impact the EEG [25].

### 2.4. EEG Recording

**Figure 4:**
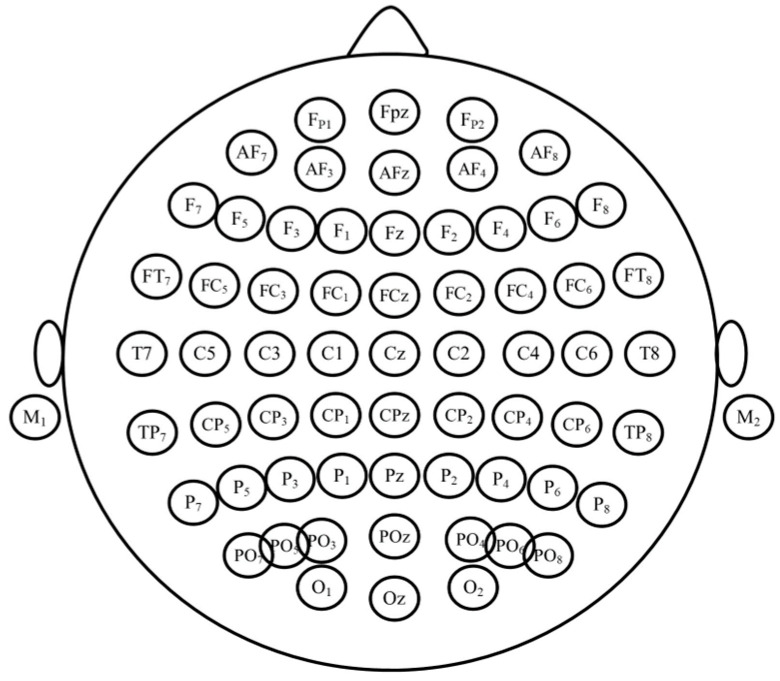
Layout of the locations of EEG channels according to the international 10–20 system.

The EEG signals were recorded using a Waveguard^TM^ 64-electrode EEG cap (manufactured by ANT Neuro, Hengelo, The Netherlands), following the 10–20 electrode placement system [40] (as shown in Figure 2). We used a TMSi REFA-8 amplifier (TMSi, Oldenzaal, The Netherlands) for EEG recording. Throughout the EEG recording, we ensured that electrode impedances remained below 10 kΩ. The EEG data were collected using Advanced Source Analysis Lab™ (ANT Neuro, Hengelo, The Netherlands) at a sampling frequency of 2048 Hz. In this study, features were extracted from the EEG data recorded from the three midline frontal, central, and parietal electrodes (lines 3, 4, and z shown in Figure 4).

### 2.5. EEG Signals Pre-Processing

The EEG data were pre-processed offline using ASA 4.10.1 and later using Python in Google Collaboratory, through which the artifacts from muscle activity and/or blinking were removed. Eyeblinks were removed through the artifact removal feature of ASA. A bandpass filter of low cut-off frequency of 0.1 Hz and high cut-off frequency of 30 Hz with a steepness slope of 24 dB/octave was used to remove the amplifier, environment, and connection noise. Artifacts with amplitude outside the region of [−100, 100] μv were also removed. Later, the EMG artifacts were removed from the signal through independent component analysis (ICA) in Python. In this study, interpolation was never required to substitute signals from a noisy channel.

### 2.6. General Machine Learning Pipeline

**Figure 5:**
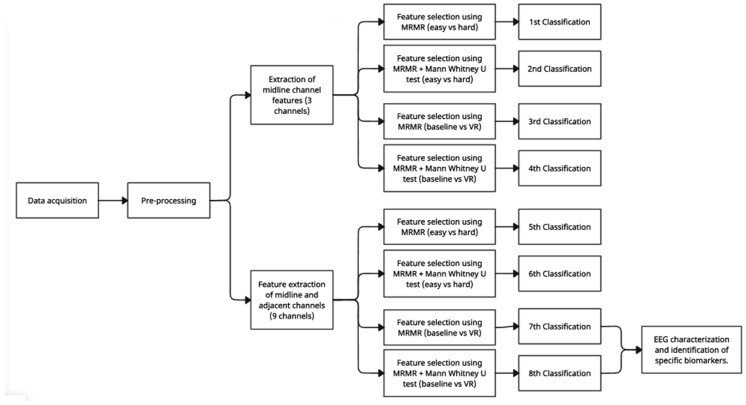
Machine learning pipeline used in this study.

All EEG signals were segmented into 4 s windows. This was performed so that in future analyses the data could be grouped to see if the level of immersion changed over time. Then, all windows are grouped and labeled according to the level of immersion for which they were recorded (i.e., three states of baseline, easy, and hard). The temporal, frequency-domain, and non-linear features were then extracted from each 4 s EEG window. According to previous work related to the use of ERPs to identify different levels of immersion during VR tasks, midline channels (Fz, Cz, and Pz) can provide relevant information about immersion levels [1,2,3,23]. In this sense, two global groups of features were generated; the first were features only extracted from the midline channels and the second were features extracted from the midline and adjacent channels (F3, F4, C3, C4, P3, P4). The reason for choosing the first group of features is for consistency with what has been previously reported in the literature [3,26]. Subsequently, feature selection was performed through two methods: one using the maximum relevance minimum redundancy (MRMR) method, and the other using the combination of MRMR with a statistical test of independence (Mann–Whitney U test). Afterwards, eight machine learning classifications were performed using different feature sets, with the first through fourth classifications using the features of the midline channels as input. The fifth through eighth classifications used the midline and adjacent channels’ features as input. The first, second, fifth, and sixth classifications differentiated the easy from hard VR states. The third, fourth, seventh, and eighth differentiated the baseline state from the difficulty. Finally, the related biomarkers were identified through EEG characterization of the best two classifiers to identify the differences between the baseline and VR states. The detailed pipeline of the data analysis and machine learning process is depicted in Figure 5.

### 2.7. Introducing the Primary Features

The features used in this study were selected primarily based on previous work that showed success in defining optimal features for ML-based approaches for the classification of EEG data for other applications [41,42]. Table 1 shows the different features that were used in this study. In total, these 10 features were used for a group of 3 and 9 channels of EEG filtered into 4 frequency bands (delta (0.2–4 Hz), theta (4–8 Hz), alpha (8–12 Hz) and beta (12–30 Hz)), resulting in the final counts of 120 and 360 for channel-band-feature trios.

### 2.8. Methods for Feature Selection

As mentioned earlier, two techniques were used for feature selection: MRMR and MRMR combined with the Mann–Witney U statistical test [46]. For the second technique, the Mann–Whitney U test was applied to the MRMR results to select the features that showed the greatest statistical difference. The MRMR approach evaluates the significance of each feature by considering two key relationships: the F statistic between each feature and the target variable or label, and the Pearson correlation between each feature and the remaining features in the data set. Consequently, a higher score indicates a greater relevance of a feature [47]. In contrast to principal component analysis (PCA), which produces principal components that are linear combinations of all original features, and linear discriminant analysis (LDA), which focuses on maximizing separability between classes based on the projection of the data on a new orthogonal basis and does not directly consider the class labels or target variable, MRMR selects a subset of original features that are directly interpretable. This can be advantageous in situations such as this study, where interpretation and understanding of the selected features (and not their combinations or projections) in relation to the problem under study are the main focus [48].

### 2.9. Classification Methods and EEG Characterization

The following classification methods were implemented and used: SGD, SVC, DT, GNB, KNN, RF, and MLP. A heuristic method was then applied to find the training hyperparameters of the models. A total of 80% of the data were used for training, and the remaining data were used to test the models. Following the classification, the channel-band-feature trios that provide the most relevant information through specific features for identifying the level of immersion are recognized and introduced as relevant markers. In this study, we evaluate the performance of the classifiers based on the accuracy percentage metric (defined as the proportion of the number of correct predictions in all predictions [49]). The parameters used for running the classification methods are summarized in Table A1 in the Appendix A to this paper.

## 3. Results

Two groups of features were generated: 120 features extracted from the midline channels and 360 features extracted from the midline and adjacent channels. The best classifier method was random forest, which obtained accuracies above 85%. With respect to the features, the most relevant channels were Fz, Cz, Pz, F3, P3, C3, F4, P4, and C4.

Table 2, Table 3, Table 4 and Table 5 show the accuracy of the tested model for each classification performed during this approach. In Table 2 and Table 4, we are using a total of 120 features (3 channels, 4 frequency bands, 10 basic features), and in Table 3 and Table 5, we are using a total of 360 features (9 channels, 4 frequency bands, 10 basic features). Table 2 and Table 3 show the accuracy percentages for classification between the easy and hard states, while Table 4 and Table 5 show the accuracy percentages for classification of baseline vs. VR state (easy and hard together). In all tables, the second column lists the accuracy percentages for the most relevant and statistically significant features obtained from the MRMR method and Mann–Whitney test, respectively, and the third column shows the accuracy percentages of the classifiers for the most relevant features resulting from only the MRMR.

All classifications were performed using different sets of data (batches) to train and test the model: all features; 10% of the total features using the MRMR method; and the features selected using the MRMR complemented by the Mann–Whitney U test. The batches for the classifications which used the midline channels’ features as input were 120 features, 12 most relevant features (according to MRMR relevance score), and 6 most relevant features (MRMR + Mann–Whitney). On the other hand, the batches for the classifications that used the features of the midline and adjacent channels were 360 features, 36 most relevant features (according to MRMR relevance score), and 20 most relevant features (MRMR + Mann–Whitney).

**Figure 6:**
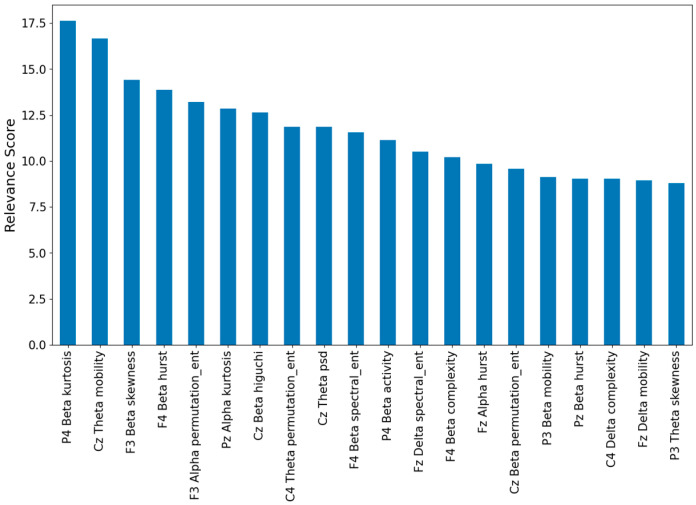
MRMR scores for the best features for baseline vs. VR classification using features of the 9 channels.

**Figure 7:**
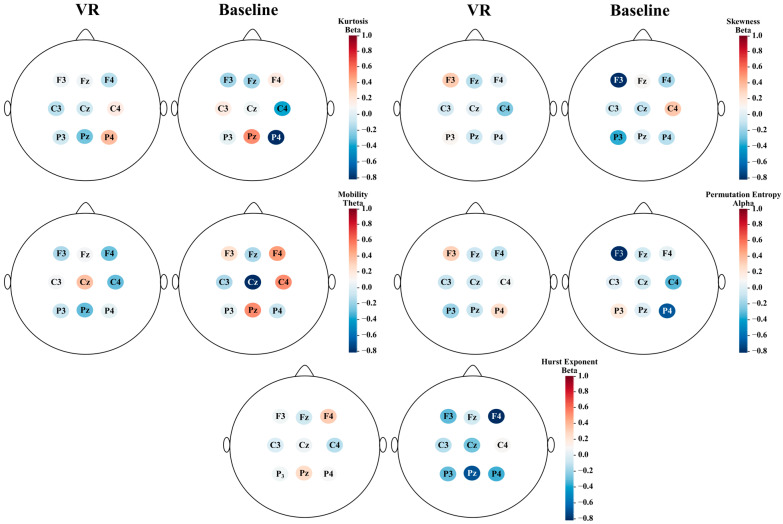
Topographic map of z-normalized mean value for most relevant features on selected electrodes.

In general, the performance of most classifiers when all features of the batch are used as input is promising. However, when the batch contains fewer features, the performance is observed to drop, as expected. This implies that by decreasing the number of features below 5%, this trend would continue, and there would be no point in performing any analysis based on the features used if the performance of the classifiers does not even exceed 75% accuracy percentage. This trend is also shown in Table 6, where the accuracy of classifiers is being reported using the best 36 features (chosen by MRMR only) and the best 5, 10, or 20 features (chosen by MRMR and Mann–Whitney together). Figure 6 shows the relevance score for the best 20 features (with the highest relevance) after applying MRMR, and Table 7 presents the *p*-value of these 20 most relevant features resulting after applying MRMR + Mann–Whitney for the fourth set of features (extracted from the midline and adjacent channels and used to classify the baseline and VR states). To better understand the association of the best features with different brain regions, Figure 7 depicts the mean of the z-normalized values of the most relevant features in different electrodes.

Based on this preliminary analysis, the EEG signal characterization and identification of possible biomarkers was accomplished using the approach that classified the baseline and VR states (easy and hard), using the features of the EEG signals of the midline and adjacent channels as input parameters.

## 4. Discussion

### 4.1. Biomarkers of Immersion in VR

To the best of our knowledge, this study is the first to use machine learning methods to classify features computed from EEG signals extracted during the performance of VR tasks. This approach was able to differentiate EEG during two levels of puzzle difficulty (easy or hard), and to differentiate the baseline state from the VR states (easy and hard together), obtaining accuracy scores above 86% and 97%, respectively.

It is important to note that the classification performance was better when more information was available (Table 3 and Table 5), which indicates that the percentage of accuracy presented here could be increased by adding more EEG channels adjacent to the midline. In addition, feature selection methods prove to be of great importance when generating more efficient classifiers without largely affecting their performance, and to perform more specific analyses on the features that provide relevant information, thus enabling the characterization of the signals under study. In this case, the combination of MRMR and the Mann–Whitney U test [50] proved to be of great help in selecting not only the most relevant features but also those that showed statistical difference between the classes (Table 7). For this reason, the order of the relevant features shown in Figure 6 is not the same as that shown in Table 7. This allowed us to obtain classifiers that still reflect promising performance using less than 5% of the total features as input (Table 6). Thus, the need for a smaller number of features implies an increase in computational efficiency when training and testing artificial intelligence models. This may prove valuable in future studies or applications that require real-time processing.

Comparing the results from Table 2, Table 3, Table 4 and Table 5 shows that while the accuracy percentage of 86% is obtained using only 10 features for classification between the baseline and VR states, such accuracy rates are obtainable only using all possible features (i.e., 360 features from all nine studied channels) for differentiating the easy and hard states, which makes a specific analysis difficult given the nature of the results obtained for this particular case. So, as a first contribution we propose possible biomarkers to differentiate between a baseline (idle) state and states related to the VR-based task (easy and hard), which is a first step towards obtaining reliable biomarkers to measure immersion.

Table 6 shows that when using the best 10 features (the first 10 features of Table 7 with the best *p*-values), five of the seven classifiers used achieved accuracy percentages higher than 85%. In the case of this particular approach, the best classifiers were SVC, RF, and MLP, with MLP being the most accurate. This may represent an opportunity for deep learning models to be included in the future to meet the same objective. Table 7 presents the most relevant final features, i.e., the features recorded in this table were the ones used to obtain the results shown in Table 6. Consequently, Figure 6 and Table 7 allow us to propose the following biomarkers to differentiate the level of immersion between a baseline state and a VR task state in a virtual reality environment: the kurtosis of the P4 and Pz channels in the beta and alpha frequency ranges, respectively, the mobility in the Cz channel in theta band, the skewness for F3 in beta band, the permutation entropy in F3 and C4 in the alpha and theta bands, respectively, the value of the Hurst exponent for F4 and Fz in beta and alpha bands, respectively, the activity in P4 in beta band, and finally, the Higuchi exponent value for Cz in beta band.

### 4.2. Association of Biomarkers of Immersion in VR and Neurophysiological Findings

A correlation between attention allocation and engagement level of immersion has been found in previous work [51]. Given the association between frontal cortex and attentional control [52], the sensitivity of features corresponding to the three frontal electrodes in the current study to the sense of immersion is unsurprising (F3 Beta skewness, F3 Alpha permutation entropy, F4 Beta hurst, and Fz Alpha hurst). This association has also been studied in the context of using auditory ERPs to investigate immersion in VR [3]. More specifically, there is a strong correlation between dorsolateral prefrontal cortex activity and planning [53], which is one of the cognitive skills involved in solving a jigsaw puzzle. The right and left prefrontal regions are associated with different functions [54,55]. While the right prefrontal cortex is more involved in strategic construction of plans, the left prefrontal cortex is more engaged in supervising the execution of the plans and control processes [53]. Fz activity has also been found to be related to the difficulty level of the task in VR [1].

This is supported by the frontal-related biomarkers of immersion found in our study (F3 Beta skewness, F3 Alpha permutation entropy, and F4 Beta hurst). As seen in Figure 7, the mean z-normalized permutation entropy of the EEG signals from the F3 channel in the beta band is relatively higher than other channels as well as the same channel in the baseline state. Permutation entropy quantifies the amount of uncertainty and unpredictability in an EEG signal [56]. Therefore, the higher permutation entropy in the F3 channel suggests that the neural activities of the left prefrontal cortex were forced to change as a result of cognitive demands related to the execution of plans to solve the puzzle. Moreover, having a relatively higher mean skewness of F3 EEG signals in the beta band (as seen in Figure 7) may be indicative of changes in the amplitude of the signals related to execution of plans. Mathematically, a highly skewed distribution may indicate the presence of outliers or rare events [57]. In contrast, Figure 7 also shows that the Hurst exponent for EEG signals recorded at F4 is relatively larger than that of the other electrodes and for the same electrode in baseline state. A greater Hurst exponent suggests more pronounced long-term correlations or persistence, where the signal tends to exhibit trends or patterns that persist over time [45]. This may be related to the association of the right prefrontal cortex with the strategic planning necessary to integrate and maintain information while solving the puzzle [54].

On the other hand, the superior parietal region has been associated with the visuospatial and visuomotor functions [58,59]. While some studies suggest that visuospatial functions should not be considered as primarily right-lateralized, the fact that the right superior parietal lobe is also involved in attention processes [53,60] might be the reason why two features related to P4 and one related to Pz appeared in the final best features, rather than a feature related to P3. The relatively higher kurtosis of EEG signals for P4 in Figure 7, compared to other electrodes, likely reflects the difference in complexity of neural dynamics underlying cognitive processes in this electrode in comparison to other ones [61].

## 5. Limitations

This is a proof-of-concept study that suggests that EEG combined with machine learning approaches may have the potential to create a real-time measure of immersion. We attempted to make the puzzle versions as similar as possible so that factors such as effort, motivation, engagement, mental exertion, cognitive demand, and interest would be similar for both puzzles; however, it is possible that these factors did vary between puzzle versions, and thus, impacted the results of the machine learning approaches.

## 6. Conclusions

To the best of our knowledge, this study is the first to introduce a machine-learning-based approach to identify markers of virtual reality immersion in EEG signals. Subjective methods of studying immersion in virtual reality do not always provide reliable results and cannot be administered in real time, while objective methods such as auditory event-related potentials have provided heterogeneous and, in some cases, contradictory results. The machine learning method used in the current study shows promising results in the test bed of a protocol that attributes immersion to the difficulty level of the task in virtual reality.

The ML approach was able to classify the EEG data collected during three different states (idle, easy, and hard) with accuracy rates of 86% and 97% for differentiating easy vs. hard difficulty states and baseline vs. VR states. Utilizing more EEG channels and features is recommended for future work in order to propose relevant biomarkers to differentiate between high and low immersion levels related to the difficulty of the VR task and cognitive load of a VR training. Similarly, in the future, we plan to include deep learning models in order to compare their performance with the classical machine learning models used in this paper.

## Figures and Tables

**Table 1:** Features used in this study.

Type of Feature	Features
Temporal	Activity (variance) [43]
	Mobility [43]
	Complexity [43]
Frequency-domain	Power spectral density (PSD)
Entropy	Permutation
	Spectral Entropy
Non-linear	Higuchi’s fractal dimension [44]
	Hurst’s exponent [45]
Statistical	Kurtosis
	Skewness

**Table 2:** Percentage accuracy for each classifier using the midline channels’ features as inputs differentiating the easy and hard puzzles as classes.

Percentage of Classification Accuracy (Easy vs. Hard) 3 Channels			
Classifier	6 Best Features	12 Features	All Features
SGD (stochastic gradient descent)	59.47	57.23	63.14
SVC (support vector classifier)	57.84	58.04	69.86
DT (decision tree)	59.27	54.79	67.01
GNB (Gaussian naive Bayes)	56.82	54.79	52.75
KNN (k-nearest neighbors)	59.27	59.06	71.69
RF (random forest)	61.30	59.06	76.37
MLP (multilayer perceptron)	59.47	60.90	73.93

**Table 3:** Percentage of accuracy for each classifier using the midline and adjacent channels’ features as input differentiating the easy and hard puzzles as classes.

Percentage of Classification Accuracy (Easy vs. Hard) 9 Channels			
Classifier	20 Features	36 Features	All Features
SGD	58.83	59.02	71.62
SVC	70.86	73.68	84.21
DT	66.73	70.11	75.19
GNB	55.08	56.20	53.76
KNN	72.74	75.75	86.09
RF	71.24	79.70	86.65
MLP	76.50	80.26	86.09

**Table 4:** Percentage accuracy for each classifier using the midline channels’ features as inputs differentiating the baseline and VR (easy and hard together) as classes.

Percentage of Classification Accuracy (Baseline vs. VR) 3 Channels			
Classifier	6 Features	12 Features	All Features
SGD	70.38	73.51	83.70
SVC	74.18	76.09	89.67
DT	73.10	72.83	81.93
GNB	67.93	68.07	75.68
KNN	74.32	75.95	87.91
RF	75.41	78.26	89.81
MLP	75.27	77.31	91.98

**Table 5:** Percentage accuracy for each classifier using the midline and adjacent channels’ features as inputs differentiating the baseline and VR (easy and hard together) as classes.

Percentage of Classification Accuracy (Baseline vs. VR) 9 Channels			
Classifier	20 Features	36 Features	All Features
SGD	85.84	87.09	93.23
SVC	86.72	88.85	96.12
DT	82.46	85.71	89.85
GNB	83.46	83.58	81.45
KNN	86.09	87.72	97.37
RF	86.34	87.22	96.87
MLP	86.22	88.35	96.49

**Table 6:** Percentage accuracy for each classifier that uses the features of the midline and adjacent channels as inputs differentiating the baseline and VR (easy and hard together) as classes.

Percentage of Classification Accuracy (Baseline vs. VR) 9 Channels					
Classifier	5 Features	10 Features	20 Features	36 Features	All Features
SGD	84.09	85.34	85.84	87.09	93.23
SVC	84.09	86.22	86.72	88.85	96.12
DT	82.46	84.84	82.46	85.71	89.85
GNB	82.08	83.58	83.46	83.58	81.45
KNN	82.21	85.71	86.09	87.72	97.37
RF	83.21	85.84	86.34	87.22	96.87
MLP	84.96	86.22	86.22	88.35	96.49

**Table 7:** *p*-value for the most relevant features based on MRMR results (Figure 6) used to obtain percentage of accuracy for baseline vs. VR in 9 channels (to obtain results in the third column of Table 6).

Feature Name	*p*-Value	Feature Name	*p*-Value
P4 Beta kurtosis	7.37 × 10^−200^	Cz Theta psd	9.82 × 10^−148^
Cz Theta mobility	3.31 × 10^−188^	Cz Beta permutation entropy	2.06 × 10^−146^
F3 Beta skewness	1.21 × 10^−185^	F4 Beta spectral entropy	6.07 × 10^−144^
F3 Alpha permutation entropy	1.91 × 10^−179^	Fz Delta mobility	1.14 × 10^−140^
F4 Beta hurst	9.89 × 10^−172^	F4 Alpha hurst	3.00 × 10^−140^
Pz Alpha kurtosis	1.02 × 10^−165^	Pz Beta activity	3.43 × 10^−137^
C4 Theta permutation entropy	2.86 × 10^−164^	Pz Alpha activity	2.33 × 10^−128^
P4 Beta activity	1.24 × 10^−161^	Fz Delta spectral entropy	6.89 × 10^−131^
Fz Alpha hurst	4.15 × 10^−157^	Pz Beta hurst	3.10 × 10^−126^
Cz Beta higuchi	3.52 × 10^−156^	F4 Beta complexity	5.28 × 10^−125^

## Data Availability

The data sets generated during and/or analyzed during the current study are available from the corresponding author on reasonable request.

## Appendix A

The following tables summarize the parameters used for running different classifiers in this study.

**Table A1:** Parameters used for running classifier differentiating the easy and hard puzzle as classes (3 channels).

Classification Parameters—(Easy vs. Hard) 3 Channels			
Classifier	6 Best Features	12 Features	All Features
SGD	alpha = 0.01	loss = log	loss = huber
	loss = squared_error	max_iter = 10	max_iter = 100
	max_iter = 100	penalty = elasticnet	penalty = elasticnet
	tol = 0.0001	tol = 10	tol = 0.0001
SVC	C = 100	C = 1	C = 1
	kernel = linear	kernel = linear	kernel = poly
	tol = 0.01	tol = 0.01	tol = 0.01
DT	ccp_alpha = 0.001	ccp_alpha = 0.001	ccp_alpha = 0.001 max_features = auto
	criterion = entropy	criterion = entropy	
	max_features = auto	max_features = auto	
GNB	var_smoothing = 1	var_smoothing = 0.01	var_smoothing = 1
KNN	leaf_size = 10	leaf_size = 10	leaf_size = 10
	metric = euclidean	metric = cityblock	metric = euclidean
	weights = distance	n_neighbors = 7	n_neighbors = 17
RF	max_depth = 10	max_depth = 5 max_features = auto	max_depth = 10
	max_features = auto		max_features = auto
	n_estimators = 500		n_estimators = 500
MLP	activation = tanh	alpha = 0.001	activation = logistic
	alpha = 0.001	hidden_layer_sizes = 500	alpha = 0.001
	hidden_layer_sizes = 500	max_iter = 5000	hidden_layer_sizes = 500
	max_iter = 5000	solver = sgd	max_iter = 5000

**Table A2:** Parameters used for running classifier differentiating the easy and hard puzzle as classes (9 channels).

Classification Parameters—(Easy vs. Hard) 9 Channels			
Classifier	20 Best Features	36 Features	All Features
SGD	alpha = 0.01	alpha = 0.01	loss = modified_huber penalty = l1 tol = 0.0001
	loss = perceptron	loss = modified_huber	
	max_iter = 10	max_iter = 100	
	penalty = elasticnet	penalty = l1	
	tol = 0.0001	tol = 0.01	
SVC	C = 100	C = 100	C = 100
	kernel = poly	kernel = linear	kernel = poly
	tol = 0.01	tol = 0.01	tol = 0.01
DT	ccp_alpha = 0.0001	ccp_alpha = 0.001	ccp_alpha = 0.001 max_features = auto
	criterion = entropy	criterion = entropy	
	max_features = auto	max_features = auto	
GNB	var_smoothing = 1	var_smoothing = 0.1	var_smoothing = 0.01
KNN	leaf_size = 10	leaf_size = 10 metric = cityblock n_neighbors = 13	leaf_size = 10
	metric = cityblock		metric = cityblock
	n_neighbors = 7		n_neighbors = 7
	weights = distance		weights = distance
RF	max_depth = 10	max_depth = 10	max_depth = 10
	max_features = auto	max_features = auto	max_features = auto
	n_estimators = 500	n_estimators = 200	n_estimators = 1000
MLP	activation = tanh	alpha = 0.001	activation = logistic
	alpha = 0.001	hidden_layer_sizes = 500	alpha = 0.001
	hidden_layer_sizes = 500	max_iter = 5000	hidden_layer_sizes = 500
	max_iter = 5000	solver = sgd	max_iter = 5000

**Table A3:** Parameters used for running classifier differentiating the Baseline and difficulty (easy and hard) as classes (3 channels).

Classification Parameters—(Baseline vs. VR) 3 Channels			
Classifier	6 Best Features	12 Features	All Features
SGD	alpha = 0.01	alpha = 0.01 loss = log max_iter = 100 penalty = elasticnet tol = 0.0001	alpha = 0.01 penalty = elasticnet max_iter = 100
	loss = squared_error		
	max_iter = 10		
	tol = 0.0001		
SVC	C = 10	C = 1	C = 100
	kernel = linear	kernel = linear	kernel = linear
	tol = 0.01	tol = 0.01	tol = 0.01
DT	ccp_alpha = 0.001	ccp_alpha = 0.001 max_features = auto splitter = random	ccp_alpha = 0.001 criterion = entropy max_features = auto
	max_features = auto		
GNB	var_smoothing = 1	var_smoothing = 1	var_smoothing = 10
KNN	leaf_size = 10	leaf_size = 10	leaf_size = 10
	metric = cityblock	metric = cityblock	metric = cityblock
	n_neighbors = 25	n_neighbors = 27	n_neighbors = 7
RF	max_depth = 7	criterion = entropy max_depth = 10 max_features = auto n_estimators = 10	max_depth = 10
	max_features = auto		max_features = auto
	n_estimators = 1000		n_estimators = 50
MLP	alpha = 0.001	alpha = 0.001 hidden_layer_sizes = 500 max_iter = 5000 solver = sgd	activation = logistic alpha = 0.001 hidden_layer_sizes = 500 max_iter = 5000
	hidden_layer_sizes = 200		
	max_iter = 5000		

**Table A4:** Parameters used for running classifier differentiating the baseline and difficulty (easy and hard) as classes (9 channels).

Classification Parameters—(Baseline vs. VR) 9 Channels					
Classifier	5 Best Features	10 Best Features	20 Best Features	36 Features	All Features
SGD	max_iter = 100 tol = 0.0001	alpha = 0.01 max_iter = 100 penalty = l1	alpha = 0.01 loss = epsilon_insensitive max_iter = 10 penalty = elasticnet tol = 0.0001	alpha = 0.01 max_iter = 10 penalty = elasticnet tol = 0.01	alpha = 0.01 max_iter = 100 tol = 0.0001
SVC	C = 100 kernel = linear tol = 0.01	C = 100 kernel = linear tol = 0.01	C = 10 kernel = linear tol = 0.01	C = 10 kernel = linear tol = 0.01	C = 10 kernel = linear tol = 0.01
DT	ccp_alpha = 0.01 criterion = entropy max_features = auto splitter = random	ccp_alpha = 0.001 max_features = auto	ccp_alpha = 0.01 criterion = entropy max_features = auto splitter = random	ccp_alpha = 0.001 max_features = auto	ccp_alpha = 0.001 max_features = auto
GNB	var_smoothing = 1	var_smoothing = 1	var_smoothing = 1	var_smoothing = 0.1	var_smoothing = 10
KNN	leaf_size = 10 metric = euclidean n_neighbors = 11	leaf_size = 10 metric = euclidean n_neighbors = 17 weights = distance	leaf_size = 10 metric = euclidean n_neighbors = 11	leaf_size = 10 metric = euclidean n_neighbors = 17	leaf_size = 10 metric = cityblock
RF	criterion = entropy max_depth = 5 max_features = auto n_estimators = 50	max_depth = 10 max_features = auto n_estimators = 1000	criterion = entropy max_depth = 10 max_features = auto n_estimators = 50	criterion = entropy max_depth = 10 max_features = auto n_estimators = 10	criterion = entropy max_depth = 10 max_features = auto n_estimators = 1000
MLP			alpha = 0.001 hidden_layer_sizes = 200 max_iter = 5000	alpha = 0.001 hidden_layer_sizes = 500 max_iter = 5000 solver = sgd	activation = logistic alpha = 0.001 hidden_layer_sizes = 500 max_iter = 5000
